# Design and synthesis of metabolic chemical reporters for the visualization and identification of glycoproteins

**DOI:** 10.1039/d1cb00010a

**Published:** 2021-02-18

**Authors:** Nichole J. Pedowitz, Matthew R. Pratt

**Affiliations:** Department of Chemistry, University of Southern California Los Angeles CA 90089 USA; Department of Biological Sciences, University of Southern California Los Angeles CA 90089 USA matthew.pratt@usc.edu

## Abstract

Glycosylation events play an invaluable role in regulating cellular processes including enzymatic activity, immune recognition, protein stability, and cell–cell interactions. However, researchers have yet to realize the full range of glycan mediated biological functions due to a lack of appropriate chemical tools. Fortunately, the past 25 years has seen the emergence of modified sugar analogs, termed metabolic chemical reporters (MCRs), which are metabolized by endogenous enzymes to label complex glycan structures. Here, we review the major reporters for each class of glycosylation and highlight recent applications that have made a tremendous impact on the field of glycobiology.

## Abstract 2

This review details a brief history of the synthesis and characterization of metabolic chemical reporters used to study glycosylation before describing recent applications and finishing with considerations and limitations of reporter molecules.

## Graphical abstract

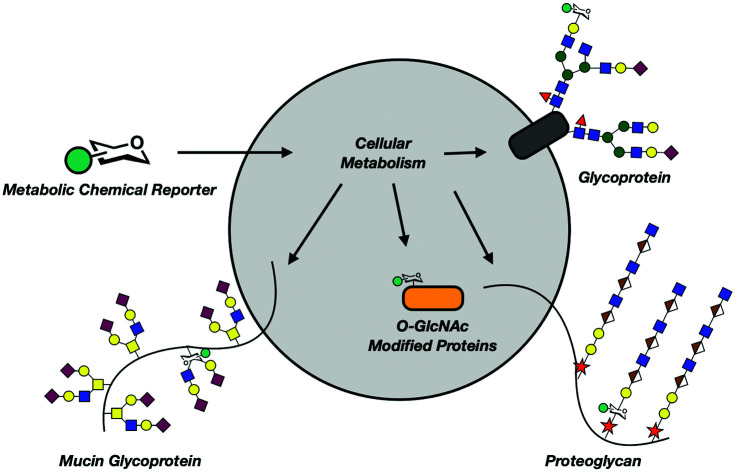

## 1. Introduction

Glycosylation is a post-translational modification (PTM) referring to the addition of one or more carbohydrate molecules to specific amino acid residues on proteins. Glycosylated proteins, termed glycoproteins, are present in all cells. The majority of glycoproteins are found on the cell surface where their oligosaccharide chain extends out into the extracellular matrix (ECM). Glycans are involved in many cellular processes including immune recognition, cellular trafficking, protein stability, and development^1^ and are associated with disease symptoms including inflammation, pathogen invasion, and malignant transformation.^2^

Our overall understanding of the structure–function relationship of glycosylation events has been hindered by a lack of practical chemical tools to study these processes. Unlike other biopolymers, oligosaccharides do not have a homogeneous arrangement of building blocks. Even within a single glycan subtype there is a huge amount of heterogeneity.^1^ Consequently, traditional biological approaches such as antibodies or affinity tags are not suitable for the study of glycans as they rely on predictable recognition epitopes not always present in complex oligosaccharide architectures. There has been some success with the development of binding lectins that recognize specific carbohydrate motifs.^3^ However, these can suffer from low binding affinity and a lack of selectivity.

Fortunately, a solution has emerged in the advancement of engineered sugar analogs termed metabolic chemical reporters (MCRs). MCRs exploit a cell's endogenous carbohydrate metabolism to convert unnatural molecules into glycosyltransferase substrates which can ultimately be added on to proteins. In this review we detail the origins of MCRs and outline the contribution bioorthogonal labeling has made in advancing the field (Sections 2 and 3). We go on to describe the first generation of MCRs, whose main goal is to deconvolute the function of the various subtypes and building blocks of glycosylation (Section 4). Knowledge gained through these studies has facilitated the use of MCRs in more complicated biological contexts and allowed us to answer questions previously beyond reach (Section 5). Finally, we conclude with a summary of the limitations of MCRs and opportunities for carbohydrate chemical biology (Sections 6 and 7).

## 2. Origin of carbohydrate MCRs

**Fig. 1:**
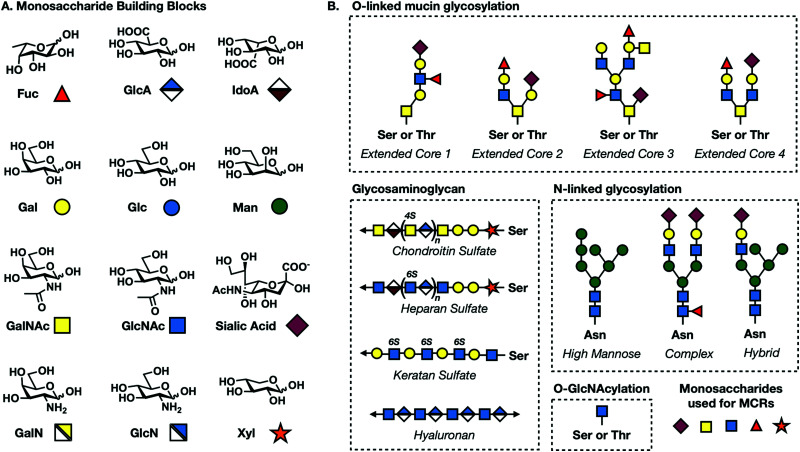
(A) Common mammalian monosaccharide building blocks and their geometric codes. (B) Core structures for *O*-linked mucin, *N*-linked, GAG, and *O*-GlcNAc glycosylation.

**Fig. 2:**
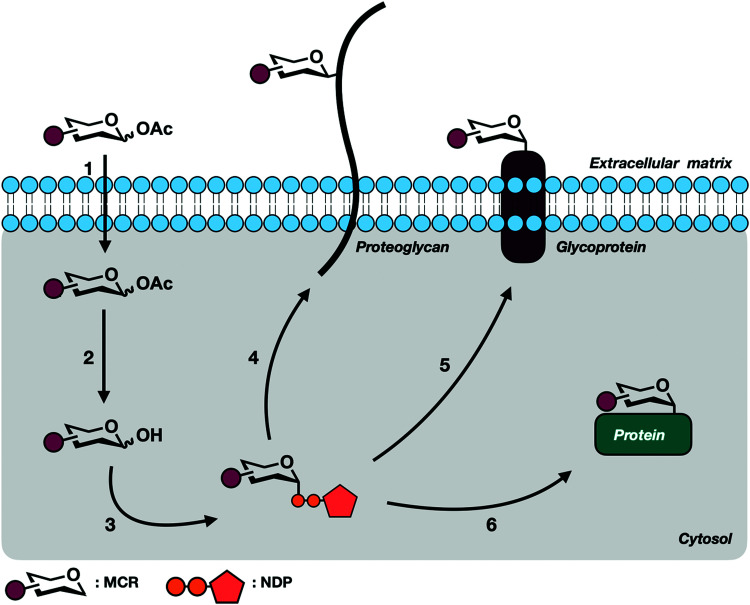
MCRs take advantage of the endogenous sugar salvage pathways that exist for most monosaccharides. (1) Per-acetylated MCRs passively diffuse across the cell membrane. (2) Promiscuous deacetylase enzymes remove acyl groups revealing hydroxides. (3) MCRs are metabolically transformed into high energy sugar donors *via* covalent bonds to NDPs. These sugar donors can then be added on to protein substrates to generate proteoglycans (4), *O*- or *N*-linked glycoproteins (5), or intracellular *O*-GlcNAc modified proteins (6).

All complex glycans are synthesized from common building blocks (Fig. 1A).^3^ The *de novo* synthesis of these building blocks relies on common metabolites such as glucose and glutamine shuttled from tightly regulated pathways. Monosaccharides can also be synthesized *via* endogenous salvage pathways where enzymes scavenge the environment for free monosaccharides and recycle these building blocks back into high energy sugar-donor substrates of glycosyltransferases (Fig. 2).^4^ Salvage pathways are uniquely adept for metabolic engineering. By starting from the monosaccharide building blocks instead of basic metabolites, there is more control over the metabolic fate of introduced biomolecules.

Reutter and coworkers recognized the potential of sugar salvage pathways.^5–7^ In pioneering work, Keppler *et al.* synthesized a series of *N*-acetylmannosamine (ManNAc) derivatives bearing one, two, or three additional carbons on the *N*-acetyl position.^6^ These analogs were successfully transformed into the corresponding sialic acid derivatives and installed on to cell surface glycoproteins. This and follow up studies established the relaxed substrate specificity of glycosyltransferases.

**Fig. 3:**
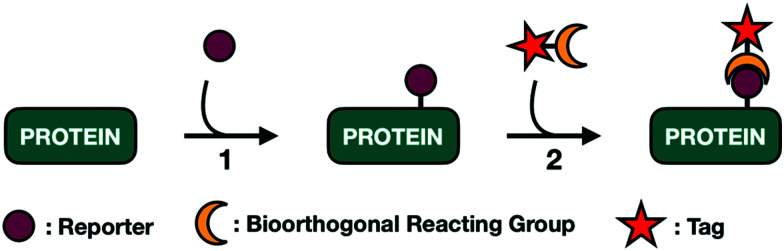
Bioorthogonal labeling of glycoproteins is a two-step process. (1) Proteins are labeled with metabolically transformed sugar analogs. (2) Probes undergo orthogonal reactions with functionalized tags allowing for downstream processing.

Soon after, the Bertozzi group pushed harder on these salvage pathways to see if their permissive nature extended to small chemically reactive groups. They recognized that appending functional groups to glycoproteins could facilitate covalent elaboration to allow for subsequent biochemical analysis. This approach relies on a two-step process (Fig. 3). First, endogenous enzymes have to incorporate unnatural sugar molecules bearing biologically inert functional groups onto protein substrates. Next, these functional groups must be paired with selective and reactive partners to form covalent adducts.

Mahal *et al.* demonstrated the first successful semi-bioorthogonal reaction using the ketone-bearing monosaccharide *N*-levulinoylmannosamine (ManLev).^8^ Though ketones are present in biological systems, they are notably absent from the cell surface where the metabolized product is deposited. Characterization of HeLa cells treated with ManLev demonstrated that it is metabolically converted into *N*-levulinoyl sialic acid (SiaLev) and incorporated onto glycoproteins. Cell-surface ketones readily react with biotin-hydrazide which can be visualized using flow cytometry. This reaction is considered only semi-bioorthogonal because there are endogenous intracellular keto-metabolites, limiting the broad use of ketones as functional handles. Nevertheless, this study was a pivotal moment in the history of bioorthogonal labeling and chemical biology as a whole, inspiring the development of countless bioorthogonal transformations.

In the 20 years since, a large number of bioorthogonal reactions have been developed. Reactions most commonly employed for the visualization of glycans are described in the next section, but this is by no means an exhaustive list. For a more in-depth description of the available range of bioorthogonal reactions, we direct readers to other comprehensive reviews.^9–11^

## 3. Bioorthogonal labeling

Bioorthogonal reactions must meet the following criteria: (1) the complimentary functional groups cannot be present in living systems, (2) these reactive groups must readily react but (3) are otherwise chemically inert, and (4) the reaction must reliably occur in aqueous environments at (5) an appreciable rate.^11^ The majority of bioorthogonal functional groups are small enough to be tolerated by carbohydrate salvage pathways, allowing chemists to outfit sugar molecules with their choice of reactive handle.

**Fig. 4:**
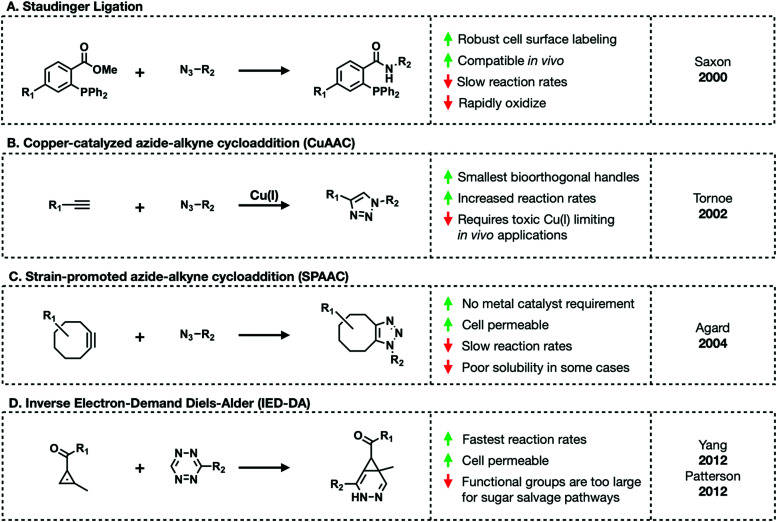
Bioorthogonal reactions commonly used for labeling glycans.

The first truly bioorthogonal reaction used in glycobiology was the Staudinger ligation.^12^ Created by Saxon and Bertozzi, the Staudinger ligation generates an amide bond between an azide and ester-functionalized triarylphosphine (Fig. 4A). These two reactive groups reliably form stable conjugates in cell lysate and live cells. An azide is small enough to be tolerated by the majority of carbohydrate salvage pathways leading to the development of diverse azide-functionalized unnatural sugars. Later work by Prescher *et al.* demonstrated that the Staudinger ligation proceeds in live mice.^13,14^ Mice treated with the sialic acid precursor, per-acetylated *N*-azidoacetylmannosamine (Ac_4_ManNAz), showed incorporation of the corresponding azido-sialic acid residues on their cell surfaces. Subsequent treatment with a phosphine-FLAG tag for western blot visualization demonstrated cell surface labeling in a live animal for the first time. Despite revolutionizing metabolic engineering, the Staudinger ligation suffers from slow reaction rates and thus require excess amounts of triarylphosphine to go to completion in reasonable lengths of time.^11^

An alternative bioorthogonal reaction is copper(i)-catalyzed azide–alkyne cycloaddition (CuAAC) or click-chemistry (Fig. 4B).^15,16^ As its name suggest, CuAAC refers to the reaction between an azide and terminal alkyne to generate stable triazole adducts. This reaction is highly selective, fast, and can proceed in aqueous environments lending to its emergence as a popular choice for *in vitro* visualization of cells and various enrichment workflows. Both azides and alkynes are small enough to be metabolized through sugar salvage pathways granting carbohydrate chemists some choice in how they functionalize their probes. However, in order to proceed at a reasonable rate this reaction requires a toxic Cu(i) catalyst impeding *in vivo* applications. Copper's toxicity can be mitigated through the tandem use of copper chelating ligands. A range of such ligands exist including TBTA, THPTA, BTTAA, and BTTES. Of these, BTTES and BTTAA are considered superior due to their increased reaction kinetics.^11^ Continued development of chelating ligands will further expand the utility of this chemistry in living systems.

Amidst the search for a copper-free substitute, Bertozzi and colleagues revisited a reaction between an azide and cyclooctyne.^17,18^ The reaction between azides and cyclooctynes, termed strain-promoted azide–alkyne cycloaddition (SPAAC), is driven by ring-strain (Fig. 4C). The cyclooctyne scaffold (OCT) has a “bent” conformation along the triple bond.^19^ This bond deformation generates strain that releases enough free energy upon 1,3-dipolar cycloaddition to proceed without a metal catalyst in aqueous environments. However, the first iteration of SPAAC suffered from the same slow rates as the Staudinger ligation. Efforts to improve the reaction rate led to the development of various functionalized cyclooctyne molecules including difluoronated cyclooctyne (DIFO) and dibenzocyclooctyne (DIBO) derivatives.^10^ These improved cyclooctynes lead to 1000× faster reaction kinetics. Unfortunately, some cyclooctynes suffer from poor solubility in aqueous environments limiting their use in biological settings.

The final major class of bioorthogonal reactions that shows great promise for *in vivo* applications are inverse electron-demand Diels–Alder (IED-DA) ligations (Fig. 4D).^20^ IED-DA reactions occur between strained *trans*-cyclooctenes (TCOs) and tetrazines. In biological settings, this reaction has the fastest kinetics of any of the previously described transformations.^21,22^ Early investigations demonstrated successful employment of this reaction in live mice. Sadly, this method relies on bulky reagents not tolerated by carbohydrate salvage enzymes. Alternative alkenes including methylcyclopropene and straight-chain alkenes are being investigated.^23–28^ Methylcyclopropenes maintain enough ring strain to readily react with tetrazines in aqueous environments. Monosaccharides modified with straight-chain alkenes have shown promise. For example, mannosamine functionalized with a butenoyl group (Ac_4_ManNBtl) successfully labeled HEK293T cells.^28^ However, in both cases these reactions are significantly slower and less efficient than those with TCOs.

In addition to the above mentioned reactions, other bioorthogonal groups have potential for widespread use in monosaccharide MCRs. One such example from the Prescher group are cyclopropenones.^29^ Cyclopropenones are small in size and react readily with triaryl phosphines making them attractive motifs for bioorthogonal chemistries. First iterations of cyclopropenones suffered from instability in aqueous environments limiting their use in biological systems. Row *et al.* stabilized these molecules by derivatizing them with electron withdrawing groups.^29^ Though there are no current example of monosaccharide analogs outfitted with cyclopropenones, they serve as an attractive alternative to established reactions.

## 4. First generation MCRs

Early advances in monosaccharide reporters aimed to develop probes selective enough to target individual subtypes of glycosylation. This goal is complicated by the fact that the major forms of mammalian glycosylation all use several of the same monosaccharide building blocks (Fig. 1A).^1^ The following section provides an introduction to each major type of mammalian glycosylation followed by a description of the MCRs that have been developed to target specific glycans or monosaccharides and probe their functions.

### 4.1. Core *N*-linked glycosylation

*N*-Linked glycans or simply *N*-glycans are branched oligosaccharides covalently bound to asparagine residues on cell surface proteins *via* a GlcNAcβ1-Asn linkage.^30,31^ The process of *N*-linked glycosylation begins when a GlcNAc residue is linked to a lipid anchoring molecule called a dolichol. Additional sugar molecules are added in a stepwise manner by various glycosyltransferases generating a common core found in all mammalian *N*-glycans consisting of two GlcNAc and three mannose residues (Fig. 1B). This pentasaccharide core is then further elaborated before enzymatic transfer of the corresponding oligosaccharide to proteins being synthesized into the Endoplasmic Reticulum. During protein folding and subsequent movement through the secretory system the glycan can be trimmed and further elaborated resulting in three extended core structures: high mannose consisting of the addition of four mannose molecules, complex consisting of the addition of GlcNAc molecules, and hybrid which is a combination of high mannose and complex (Fig. 1B). *N*-Linked glycans can be transferred onto proteins with the basic amino acid motif Asn-X-Ser/Thr with X being any amino acid except proline.

*N*-Linked glycosylation occurs co-translationally while a nascent peptide is being synthesized in the ER.^30^ The extended oligosaccharide structures are recognized by lectin chaperones that aid in proper protein folding. Proteins that are not folded properly undergo specific *N*-glycan trimming, generating truncated oligosaccharides which mark the protein to be retrotranslocated into the cytoplasm and degraded. Protein folding and other important functions of *N*-linked glycosylation have been shown to be critical for normal cell functioning. Misregulation of this form of glycosylation has been implicated in metabolic disorders and some cancers.

The design and synthesis of selective MCRs for *N*-linked glycans has proven to be challenging due to the overlap of common monosaccharide building blocks and inefficient turnover by salvage pathway enzymes. One successful example comes from the Bertozzi group who incorporated GlcNAlk and GlcNAz onto *N*-linked glycans in yeast.^32^ To do so, they engineered a strain of *Saccharomyces cerevisiae* to be dependent on exogenous GlcNAc. Their reliance on an engineered strain of yeast suggests that salvage pathways that feed to *N*-linked glycan synthesis compete ineffectively with endogenous UDP-GlcNAc. However, the results indicate that GlcNAc-based MCRs could be successfully incorporated on to *N*-linked glycans in mammalian cells. Unfortunately, both of these reporters have been shown to be incorporated as intracellular *O*-GlcNAc modifications. To date, there are no reporters that can selectively label *N*-linked glycans’ core pentasaccharide structure.

### 4.2. Mucin *O*-linked glycosylation

Mucin *O*-linked glycosylation is the most abundant form of glycosylation.^1,33^ It is characterized by the addition of GalNAc to Ser and Thr residues on cell surface proteins that can then be further elaborated. GalNAc is metabolically transformed into the high energy sugar-donor UDP-GalNAc. It is then recognized by a family of enzymes known as polypeptide *N*-acetyl-α-galactosaminyltransferases (ppGalNAcTs)^34^ and added to proteins in the Golgi apparatus. Additional sugar molecules can continue to be added *via* C3 or C6 linkages to the core GalNAc residue generating one of six core structures, four of which are expressed broadly in mammalian cells (Fig. 1B).^35^ These can be further elaborated into complex and heterogenous linear or branched oligosaccharide trees. Mucin *O*-linked glycans serve a variety of functions including promoting or repelling cell-surface interactions, protecting against infection, maintaining cellular homeostasis, and serving as a barrier for cells from physical or chemical damage.^36^

**Fig. 5:**
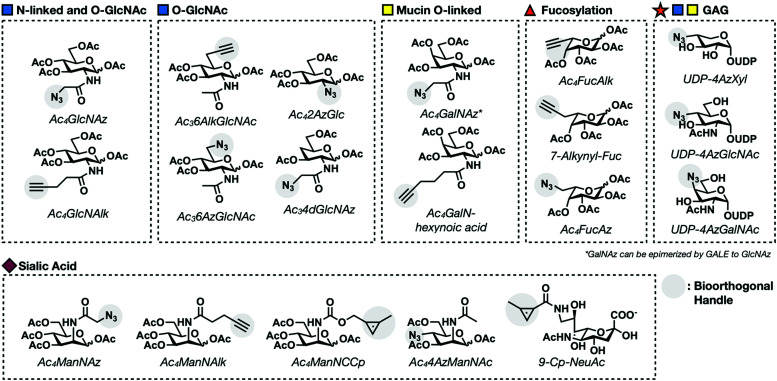
Examples of first generation metabolic chemical reporters for different glycosylation events.

Mucin MCRs have primarily been designed to target the initial GalNAc residue present in all mucin *O*-linked glycans. The first example came from Hang *et al.* who synthesized a GalNAc analog functionalized with an azide tag on the *N*-acetyl position, *N*-azidoacetylgalactosamine (GalNAz, Fig. 5) and demonstrated that it can label cells.^37^ CHO cells were treated with Ac_4_GalNAz and reacted with phosphine-FLAG. Following the reaction, cells were labeled with FITC-conjugated anti-FLAG and analyzed *via* flow cytometry. GalNAz fluorescence was 30-fold higher than background fluorescence demonstrating efficient incorporation into cell surface glycoproteins. Two other reporters, Ac_4_2AzGal and Ac_3_6AzGalNAc, showed no significant fluorescence labeling over background suggesting that azide functionalities at these positions are not tolerated by the GalNAc salvage pathway. GalNAz labeling was further characterized through competition experiments. Co-treatment with 5 mM GalNAc completely removed cell surface labeling demonstrating that GalNAz was being metabolically incorporated through the GalNAc salvage pathway. Competition with GlcNAc resulted in partial inhibition of labeling consistent with UDP-GlcNAc's potential to be epimerized to UDP-GalNAc by the enzyme UDP-glucose 4-epimerase (GALE).

Zaro *et al.* continued characterizing GalNAz by examining its incorporation into known glycoproteins, to determine that GalNAz is preferentially incorporated into mucin *O*-linked glycans over *N*-linked glycans.^38^ GlyCAM-IgG, a glycoprotein with both *N*-linked and *O*-linked glycosylation, was expressed in the presence of Ac_4_GalNAz. The IgG domain was isolated with Sepharose beads and the enriched protein was subjected to CuAAC with an alkyne-fluorophore tag. In-gel fluorescence scanning showed robust labeling by GalNAz. Subsequent treatment with PNGase F, an enzyme that removes *N*-linked glycans, exhibited minimal decrease in signal, indicating that the majority of GalNAz residues are incorporated into mucin *O*-linked glycans. Despite achieving biased labeling for mucin *O*-linked over *N*-linked glycans, GalNAz has been shown to be incorporated as an *O*-GlcNAc modification.^35^ UDP-GalNAz serves as a substrate for GALE and can be epimerized into UDP-GlcNAz. More details of GalNAz as an *O*-GlcNAc MCR can be found in the O-GlcNAc modification Section (4.5). Dube *et al.* evaluated GalNAz labeling in mice.^40^ Ac_4_GalNAz was injected into live mice for 7 days. Following treatment, their organs were harvested and GalNAz incorporation was evaluated using the Staudinger ligation with phosphine-FLAG. GalNAz labeling varied depending on the cell-type. Of particular interest was inconsistent labeling between different types of spleen cells. Splenocytes could be separated into two distinct populations based on labeling efficiency. Further investigation determined that Ac_4_GalNAz has higher incorporation in B-cells than T-cells, a property that can be leveraged to target specific splenocyte populations.

Despite the success of GalNAz as a global mucin *O*-linked labeler, assessing the specific activity of individual ppGalNAcT isozymes has remained a major challenge. Recently, a collaboration between the Schumann and Bertozzi groups has started to address this limitation by developing bump–hole sugar–protein pairs.^41–44^ Their strategy involves synthesizing GalNAc analogs with extended carbon chains ending in a bioorthogonal group on the *N*-acetyl position (Fig. 5). The additionally carbons add a “bump” that prevents endogenous ppGalNAcTs from turning over the corresponding UDP sugar-donor. These reporters were partnered with mutated ppGalNacT enzymes with a complementary “hole” in the active site. Initial characterization *in vitro* proved mutant ppGalNAcT enzymes selective modify peptides with UDP-GalNAc bearing a hexynoic acid substitution on the *N*-acetyl position in the presence of wild-type enzymes.^41^

Moving this system into cells is complicated by two factors: mutant ppGalNAcTs must properly function and the bulky GalNAc analogs rely on other enzymes to be converted into UDP sugar-donors. Fortunately, mutant ppGalNAcTs were shown to function properly when stably introduced in HepG2 cells. To circumvent potential issues with the biosynthesis of bulky UDP-GalNAc, the Bertozzi group synthesized a cell permeable protected GalNAc-1-phosphate precursor which was able to bypass the majority of enzymes in the GalNAc salvage pathway leaving only the final step catalyzed by the enzyme AGX1.^42^ Mutant AGX1 that can tolerate bulky substituents on the *N*-acetyl position has been developed by the Kohler lab.^45^ Introducing these mutants resulted in the successful synthesis of bumped UDP-GalNAz sugar-donors. This engineered system successfully incorporates reporters on cell surface mucin-type glycans, introducing the first gain-of-function strategy toward deconvolution ppGalNAcT functions. However, the need to generate double mutant cell lines expressing exogenous AGX1 and ppGalNAcT is as a major limitation for implementing this strategy in more biologically relevant settings.

### 4.3. Sialylation

Sialylation is the addition of members a family of 9-carbon monosaccharides called sialic acids on to cell surface glycoproteins.^1^ This addition terminates further elongation of the glycan chain. Sialic acid residues are known to have important roles in mitigating cell–cell, cell–glycan, and cell–pathogen interactions. This is due in part to the fact that sialic acids are negatively charged at neutral pH. Their electronegative character also contributes to protein stabilization and ion binding.

As mentioned above, sialic acid metabolism was the first to be interrogated using engineered monosaccharides with the invention of ManLev.^8^ Success with ManLev inspired Bertozzi and others to continue developing sialic acid reporters with increasingly selective functionality. The first truly bioorthogonal MCR was created by Saxon and Bertozzi who applied the Staudinger ligation to a reaction between the functionalized ManNAc analog, Ac_4_ManNAz, and a phosphine tag.^12^ Ac_4_ManNAz is metabolized, forming the corresponding sialic acid bearing an azide, SiaNAz. Incorporation into the cell surface was visualized using the phosphine-FLAG tag followed by flow cytometry. Prescher *et al.* demonstrated that Ac_4_ManNAz successfully labels live mice.^13^ This is the first example of MCR incorporation in a live animal, inspiring the application of Ac_4_ManNAz to visualize more complicated disease models such as cancer. Increases in sialic acid on the cell surface has been shown to correlate with malignancy of cancer types. Toward this goal, Neves *et al.* investigated the possibility that labeling with Ac_4_ManNAz could serve as a noninvasive way to monitor tumor progression.^46^ Mice with lung carcinoma xenografts were injected intraperitoneally with Ac_4_ManNAz and labeled *in vivo* with phosphine tags. Xenograft tissue had elevated fluorescence signal attributed to sialic acid labeling compared to healthy tissue indicating selective labeling.

Azide-containing unnatural sugars were the first to be characterized. However, the advent of CuAAC click-chemistry reactions between azide and terminal alkynes open the possibility for reversing the orientation of reporter and tag functionalities as alkynes are small enough to transduce most carbohydrate salvage pathways. Wong and coworkers developed an alkyne-modified ManNAc probe termed *N*-pentynoylmannosamine (Ac_4_ManNAlk), reasoning that it would be converted to the corresponding SiaNAlk sugar and displayed on the cell surface.^47^ Treatment of Hep3B cells with Ac_4_ManNAlk followed by CuAAC with a fluorogenic azido-hydroxycouramin probe resulted in robust labeling over background. Switching the orientation of functionalized handle on the probe and tag is advantageous. Even the most efficient bioorthogonal reactions require excess tag to go to completion. Though azides and alkynes are both thought to be abiotic, alkynes exhibit some amount of reactivity in biological environments leading to higher background labeling when using excess alkyne-tag. Therefore, it is worthwhile to generate alkyne-reporters to ensure that the reagent used in excess is the completely inert azide.

MCRs for sialylation so far have been functionalized on solely on the *N*-acetate of the metabolic precursor ManNAc resulting in sialic residues functionalized at the C5 position. To investigate the efficiency of functionalizing other positions, Hackenberger and coworkers synthesized a C4-substituted ManNAc analog, Ac_3_-4-azido-ManNAc (Ac_3_4AzManNAc).^48^ Ac_3_4AzManNAc is converted to sialic acid molecules functionalized at the C7 position which can be added to cell surface glycoproteins to label cells. Characterization of Ac_3_4AzManNAc labeling showed that it selectively labeled *O*-linked glycans demonstrating an early example of structural changes biasing the metabolic fate of functionalized sugars. Recently, sydnone functionalized sialic acid MCRs have emerged as an alternative to traditional azide bearing reporters. Chinoy *et al.* synthesized sialic acid derivatives with sydnones on either the C5 or *N*-acetyl position, termed Neu5SydCl and Neu9NSydCl respectively.^49^ Interestingly, Neu5SydCl was not metabolically efficiently transferred on to protein substrates while Neu9NSydCl showed robust labeling. In addition, Neu9NSydCl could only be tolerated a few STs indicating that Neu9NSydCl labeling could differentiate among various types of sialosides.

### 4.4. Fucosylation

l-Fucose is a 6-deoxyhexose found on the termini of *N*-linked and *O*-linked glycans (Fig. 1A and B). Structurally, fucose distinguishes itself from other 6-carbon sugars due to its lack of a hydroxyl group at the 6-position and l-configuration. The addition of fucose is catalyzed by a class thirteen of enzymes known as fucosyltransferases (FucTs). FucTs transfer fucose as a high energy sugar-donor, GDP-fucose. Fucosylation has been shown to play a role in a variety of cellular functions including cell–cell and cell-pathogen interactions as well as being elevated during development.^50^

Researchers have exploited the fucose salvage pathway to incorporate chemically reactive groups on cell surface glycans.^4^ The first example of a fucose MCR came from the Sawa *et al.* as an azide-bearing fucose analog, Ac_4_FucAz (Fig. 5).^51^ Ac_4_FucAz enters the fucose salvage pathway and is converted to GDP-FucAz. From here, GDP-FucAz is transferred onto cell surface fucosylated glycans. Ac_4_FucAz incorporation can be visualized using CuAAC labeling with an alkyne-based fluorescence tag. Concurrently, Rabuka *et al.* synthesized fucose analogs with azide substitutions at the C2, C4, and C6 positions (Fig. 5).^52^ 2- and 4-Azido fucose are not tolerated by the fucose salvage pathway and therefore not metabolically incorporated onto glycans. 6-Azido-fucose was successfully incorporated but showed high levels of cytotoxicity. Hsu *et al.* addressed this limitation by switching the orientation of the probe and tag, generating FucAlk probes and observed reduction in cytotoxicity.^47^

All of the aforementioned fucose MCRs suffer from low levels of metabolic labeling in mammalian cells. This limitation prompted the transition to zebrafish to determine the limiting step in fucose metabolism. The first example of this came from Wu and coworkers who synthesized UDP-FucAlk and injected it directly in to zebrafish embryos.^53^ To test for incorporation, labeled embryos were subjected to CuAAC with a biocompatible Cu(i)-complex, bis(*tert*-butyltriazolyl) ligand (BTTES) which serves as a copper chelating agent, preventing toxic copper(i) from passing through the cell membrane. 2.5 Hours post injection, fucosylated glycans could be visualized in the enveloping layer indicating that limitations in fucose metabolism occur during the initial steps of the fucose salvage pathway and not the transfer of GDP sugar-donors. Okeley *et al.* were able to improve FucAlk's labeling efficiency using a rate-accelerating, chelating azide-assisted CuAAC reaction leading to the discovery that FucAlk also acts as a metabolic inhibitor of fucosylation.^54^ Haltiwanger and coworkers capitalized on the inhibitory effects of fucose MCRs to attenuate Notch signaling in zebrafish.^55^ Specifically, Zebrafish embryos treated with FucAlk prevented Notch signaling resulting in the prevention of T-cell differentiation.

Building on these findings, Kizuka *et al.* generated a fucose MCR with an extension off of the C5 position to give 7-alkynyl-fucose, eliminating these inhibitory affects (Fig. 5).^56^ Subsequent analysis showed that FucAlk is selectively incorporated into the core position of *N*-linked glycans. Recently, Kizuka and coworkers compared the labeling efficiency of FucAlk and 7-alkynyl-fucose (Fig. 5) to learn more about fucose metabolism.^57^ A comparison of these reporters across different cell lines showed differential incorporation that was both cell and protein dependent. Subsequent *in vitro* assays demonstrated that tolerance of FucTs dictates the labeling efficiency of fucose analogs. Cell lines expressing FucTs with larger binding pockets had a higher tolerance for the additional carbon present in 7-alkynyl-fucose and resulting in higher overall labeling efficiency.

### 4.5. *O*-GlcNAc modification

*O*-GlcNAcylation refers to the addition of the monosaccharide *N*-acetylglucosamine (GlcNAc) to serine and threonine residues of intracellular and mitochondrial proteins (Fig. 1B).^58–60^*O*-GlcNAc distinguishes itself from other forms of glycosylation in three ways; (1) *O*-GlcNAc substrates are primarily cytosolic, (2) *O*-GlcNAcylation is dynamic, and (3) GlcNAc residues are not further elaborated. The cycling of *O*-GlcNAc is catalyzed by two enzymes, OGT and OGA, which add and remove the modification respectively.^61,62^ The majority of GlcNAc molecules are synthesize from glucose *via* the hexosamine biosynthetic pathway (HBP) to be transformed into UDP-GlcNAc. Because GlcNAc is primarily synthesized from the HBP, overall modification levels are sensitive to the amount of circulating glucose. This allows *O*-GlcNAcylation to serve as a nutrient sensor.

Both its dynamic nature and sensitivity to circulating metabolites establishes *O*-GlcNAcylation as a vital regulator of numerous cellular functions including gene expression, signal transduction, stress response, and protein stability. Taken together, these functions solidify *O*-GlcNAcylation as essential to normal growth and development in nearly all multicellular organisms. This is further demonstrated by experiments showing that genetic knockout of OGT is embryonically lethal in Drosophila and mice and tissue specific knockouts of OGT in T-cells causes apoptosis.^63–66^ Further, loss of OGT in neurons is associated with neurodegeneration in mice. *O*-GlcNAc levels are consistently seen to be upregulated in cancer and diabetic patients and lowered in neurodegenerative disorders.^67–71^

Efforts have been made by our lab and others to develop a selective *O*-GlcNAc reporters. This is challenging because UDP-GlcNAc is a substrate for other glycosyltransferases and is incorporated into both *N*-linked and *O*-linked glycoproteins. Hang and Vocadlo *et al.* developed the first *O*-GlcNAc reporters termed Ac_4_GlcNAz and Ac_4_GalNAz (Fig. 5).^37,39^ These two molecules are C4 epimers that can be interconverted by the enzyme UDP-glucose 4-epimerase (GALE). They synthesized UDP-GlcNAz and all upstream metabolites of the GlcNAc salvage pathway in order to demonstrate that all relevant enzymes including OGT could tolerate the azide analogs *in vitro*. Additionally, they showed that OGA cleaves GlcNAz at similar rates as GlcNAc indicating that these reporters do not perturb *O*-GlcNAc dynamics. Treated Jurkat cells showed that these reporters could label the constitutively *O*-GlcNAc modified protein family nucleoproteins. A later study by Boyce *et al.* aimed to investigate the cross talk between the GalNAc and GlcNAc salvage pathways.^72^ They showed that Ac_4_GalNAz did label nucleocytoplasmic proteins and this labeling increased in cells overexpressing OGT. This served as confirmation that GALE was able to interconvert UDP-GalNAz to UDP-GlcNAz. The same study also showed that UDP-GalNAz had superior labeling to GlcNAz presumably due to GalNAz having a more tolerable salvage pathway allowing for more efficient conversion of Ac_4_GalNAz to UDP-GalNAz.

Zaro *et al.* improved labeling by generating Ac_4_GlcNAlk, an alkyne substituted reporter that can be labeled with CuAAC (Fig. 5).^38^ GlcNAlk showed robust labeling with an improved signal-to-noise ratio compared to GlcNAz. This study further demonstrated that both GlcNAlk and GlcNAz were incorporated and removed as similar rates indicating that GlcNAlk does not affect modification dynamics. Unfortunately, both GlcNAz and GlcNAlk are not specifically incorporated on *O*-GlcNAcylated proteins. Enrichment of lysates labeled with these reporters identified the reporter protein GlyCAM-IgG which is known to be have *N*- and *O*-linked glycosylation. Removal of *N*-linked glycans significantly reduced labeling of both GlcNAz and GlcNAlk, further demonstrating that they are incorporated into *N*-linked glycans.

In an effort to generate an MCR specific for *O*-GlcNAcylation, we sought to exploit OGT's promiscuity to make more exotic sugar analogs. Inspired by work demonstrating that UDP-6-azido-6-deoxy-GlcNAc was a substrate for OGT *in vitro*, we synthesized the reporter Ac_3_6AzGlcNAc (Fig. 5).^73,74^ Treatment of cells with 6AzGlcNAc showed similar labeling patterns and intensity compared to cells treated with GlcNAz demonstrating that the GlcNAc salvage pathway tolerates modifications made on the C6 position. Enrichment of cell lysate treated with 6AzGlcNAc pulled-down known *O*-GlcNAc modified proteins NEDD4, pyruvate kinase, nucleoporin 62, and FoxO1 but not GlyCAM-IgG demonstrating the selective incorporation into *O*-GlcNAcylation.

The success of Ac_3_6AzGlcNAc lead us to seek to improve the labeling efficiency by generating an alkyne derivative also modified at the C6 position: Ac_3_6AlkGlcNAc (Fig. 5).^75^ Fluorescent labeling with 6AlkGlcNAc showed a similar banding pattern and intensity compared to 6AzGlcNAc but with a better signal-to-noise ratio. Cells treated with 6AlkGlcNAc and then submitted to a proteomic workflow identified caspase-3 and caspase-8 as *O*-GlcNAc modified proteins. Both were confirmed *via* enrichment and western blotting. It was ultimately determined that caspase-8's modification sites were adjacent to its cleavage/activation site. Through a series of experiments, we were able to show that *O*-GlcNAc modifications at these sites blocks cleavage and therefore activation, preventing the activation of the caspase cascade.

To test the breadth of OGT's promiscuity, two additional reporters have been developed; Ac_4_2AzGlc and 4-deoxy-GlcNAz (Ac_3_4dGlcNAz) (Fig. 5).^76,77^ Both are successfully turned over by OGT and appear to be selective for *O*-GlcNAcylation. However, the per-acetylated form of 2AzGlc, Ac_4_2AzGlc, is toxic in cells treated for longer periods of time (200 mM for 16 hours). This was consistent with work published by the Yarema lab showing that per-acetylated *versus* partially deprotected MCRs have different metabolic fates and exhibit different toxicities.^78^ To address this, we removed the 6-*O*-acetyl group to form Ac_4_2AzGlc which reduced the toxicity. 4dGlcNAz was proposed to be more selective for *O*-GlcNAcylation over *N*- and *O*-linked glycosylation because linkages at the C4 position of GlcNAc residues are important for extension of cell surface glycans. Characterization 4dGlcNAz showed selective labeling of intracellular *O*-GlcNAc modified substrates.^77^ However, 4dGlcNAz is resistant of removal by OGA.^79^ While this increases labeling efficiency, perturbing *O*-GlcNAc dynamics limits the physiological relevance of cellular and *in vivo* experimental results.

### 4.6. Glycosaminoglycan

Glycosaminoglycans (GAGs) are linear polysaccharides consisting of repetitive disaccharide building blocks. GAGs are covalently bound to protein substrates to form glycosylated proteins termed proteoglycans.^80–82^ GAGs are classified into four broad subtypes; hyaluronan (HA), keratin sulfate (KS), chondroitin sulfate/dermatan sulfate (CS/DS), and heparin/heparan sulfate (HS) (Fig. 1B). All GAG subtypes share a characteristic pattern of alternating hexosamine and uronic acid subunits with the exception of KS which alternates between hexosamine and galactose. Subtypes differ in their linkages to proteins. KS polysaccharides are linked to underlying *N*-linked or mucin *O*-linked glycans while CS/DS and HS chains are attached to proteins at serine residues through a unique core linkage. First, a xylose monosaccharide is attached followed by step-wise addition of two galactose and one glucosamine residue to generate a tetrasaccharide core with the following linkages: GlcAβ(1–3)Galβ(1–3)Galβ(1–3)Xylβ-serine. CS/DS subtypes distinguish themselves from HS through the addition of a GlcNAc residue following this core structure. HS proteoglycans are characterized by GalNAc. Proteoglycans are ubiquitous, having been identified in almost all mammalian cell types. Proteoglycans are secreted into the ECM and either inserted in the plasma membrane or stored in secretory granules.^83^ GAGs can be further modified through the addition of sulfate groups on GlcNAc or GalNAc residues resulting in an increased negative charge^80^ which facilitates interactions with proteases,^84^ growth factors,^84^ cytokines,^85^ lectins,^85^ and structural proteins.^86^

To date, there are no MCRs that label a fully formed proteoglycan. However, a few reporters that are incorporated as modified core monosaccharides leading to premature truncation of the GAG polysaccharide chain but allowing for downstream tagging. One example comes from Linhardt's lab in the synthesis of UDP sugar-donors of the hexosamine subunits, 4AzGlcNAc and 4AzGalNAc (Fig. 5).^87^ Linhardt was able to incorporate these probes onto HS and HA polysaccharide chains *in vitro*. Unfortunately, azide modifications on the C4 position of hexosamine sugars are not tolerated by salvage pathway enzymes, limiting cellular applications for these MCRs.^88^ In a similar approach, the Bertozzi lab generated C4 analogs of xylose by synthesizing the UDP donor-sugar of 4-azido-4-deoxy-xylse (4AzXyl) (Fig. 5).^89^ To bypass salvage pathway limitations, UDP-4-AzXyl was directly injected into zebrafish embryos resulting in its incorporation into the core position of CS/DS and HS proteoglycans preventing GAG extension but allowing for tagging and analysis of the abundance and distribution proteoglycans of zebrafish cells at different stages of development.

## 5. Next generation MCRs

In recent years, efforts have been made toward probing glycoproteins in more complicated biological contexts. The following section provides examples of the diverse applications of MCRs and how they have contributed to major breakthroughs toward a complete understanding of global glycosylation events.

### 5.1. Cell-specific labeling

The exploratory phase of glycan engineering mainly took place in homogenous cell populations. Preliminary MCR investigations into live animals saw reporters readily incorporated into a myriad of cell types to indiscriminately label tissue.^13^ Systemic labeling of whole organisms offers valuable information about glycan density and distribution. However, cell- and tissue-specific labeling in heterogenous cellular environments expands the utility of reporter molecules.^90^ For example, selective incorporation of azide-clad monosaccharides on to the surface of cancer cells enables orthogonal conjugation of an anti-cancer drug for proximity based targeted drug delivery. The majority of work toward cell-specific labeling falls into two categories: selective de-caging and liposomal delivery.

Different types of cells express different proteins. Cancer cells in particular have unique expression profiles with many protease enzymes constitutively overexpressed. Researchers have exploited this feature to unleash caged probes into complex biological environments. Bertozzi and coworkers reported the first example of a caged sugar MCR.^91^ Ac_4_ManNAz was modified with a linker attached to a peptide sequence masking the C6-hydroxyl group. The peptide sequence can be cleaved by a prostate-specific antigen protease (PSA). Once released, the de-caged mannose analog permeates proximal cell membranes leading to selective labeling of PSA-expressing cells. Caged Ac_4_ManNAz was also used by Wang *et al.* in a metastatic breast cancer model.^92^ Here, Ac_4_ManNAz was caged at the anomeric position *via* an ether bond linked to an enzyme cleavable moiety. This caged MCR was designed to require sequential cleavage events to be released. Histone deacetylase and Cathepsin L must both be present, adding an additional level of rigor to ensure cell specific activation. This process successfully labeled triple-negative breast cancer; a form notoriously hard to treat in clinical settings.

**Fig. 6:**
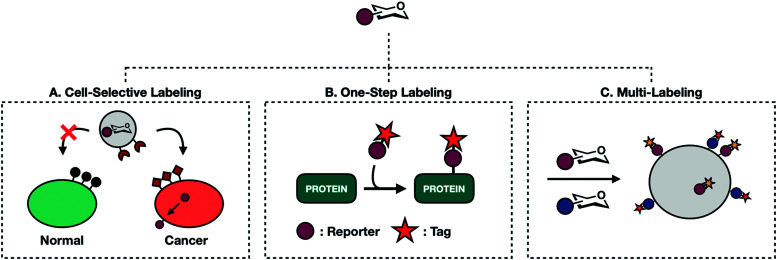
Second generation MCRs expand their applications. (A) Encapsulating reporters in liposomes allows for targeting of a specific class of cells of tissues. (B) One-step labeling eliminates secondary incubation with tags allowed for direct detection of reporters on protein substrates. (C) The diverse range of bioorthogonal reactions allows for multi-labeling experiments where two bioorthogonal reactions can be performed concurrently without risking cross-labeling.

The caging strategy's major limitation is its reliance on endogenous proteases. This restricts targetable cells to those with unique or overexpressed proteases. Targeting unique proteases also demands the synthesis of different caged monosaccharides for each protease/sugar-analog pairing. In an effort to develop a more general approach, Chen and coworkers have adapted well defined liposomal delivery systems for MCR delivery (Fig. 6A).^93–95^ The first iteration involved encapsulating 9AzSia in liposomes functionalized with folate generating a complex termed f-LP-9AzSia.^93^ Folate receptors (FRs) are overexpressed in many epithelial tumor tissues including ovarian, breast, lung, and colorectal cancers. Treatment in HeLa cells, an FR expressing cell line, followed by CuAAC assisted with ligand BTTAA conjugation to a fluorescent probe exhibited concentration dependent cell surface labeling. When administered in a mixed population of FR expressing (FR^+^) and non-expressing cells, f-LP-9AzSia discriminately labeled FR^+^ cells. Chen has since been able to successfully employ this strategy to discriminately label organ tissues and mouse brain cells *in vivo*.^94,95^

### 5.2. One-step labeling

Traditional MCR labeling relies on a two-step procedure where reporters are first installed onto cellular substrates followed by a reaction with a bioorthogonal probe (Fig. 3). The product is a covalent tertiary structure composed of substrate-reporter-tag. Efficient labeling therefore relies on the rate of both metabolic incorporation and the reaction between reporter and tag. To simplify this process, efforts have been made to generate one-step labeling mechanisms allowing for the direct visualization of unnatural glycans without further conjugation (Fig. 6B). Toward this goal two different techniques have emerged: Raman scattering and pre-conjugated sugar analogs.

Raman spectroscopy can directly detect metabolic probes on the cell surface. Raman spectroscopy uses Raman scattering which can detect specific vibrational wavelengths of molecules of any size. Each type of bond has a specific signal, analogous to a vibrational fingerprint.^96^ Chemical reporters containing abiotic functional groups introduce distinct vibrational patterns into cellular environments. Conveniently, the most commonly used bioorthogonal functional groups, azides and alkynes, exhibit vibrational signatures in the Raman-silent region of a cell (between 1800–2800 cm^−1^) allowing the signal from these molecules to rise above noise from cellular milieu. The major limitation of Raman spectroscopy is the inherently low signal produced with traditional MCRs.^96^ Passive Raman spectroscopy relies on an accumulation of probes in a concentrated location. Given that one goal of glycan engineering is to reflect endogenous activity of glycoproteins and the fact that monosaccharides are generally dispersed throughout the entirety of the cell, they cannot be imaged based on a single concentration point. Chen and coworkers addressed this limitation with surface-enhanced Raman spectroscopy (SERS).^97^ SERS employs gold plasmonic nanoparticles (AuNPs) conjugated with 4-mercaptophenylboronic acids (MPBA). MPBA has a high affinity for sialic acids. Cells treated with Ac_4_ManNAlk followed by incubation with MPBA-AuNPs gave modified sialic acid residues that could be easily distinguished from their endogenous counterparts using dark field microscopy. This workflow can transition back to a one-step process by making MCRs whose functionalized handle is a small Raman reporter. Unfortunately, metallic nanoparticles are required to boost Raman signal, limiting their use for intracellular imaging.

An alternative approach is to generate pre-conjugated sugar analogs outfitted with fluorescent or biotinylated tags. These can be directly incorporated on to cell surface and intracellular glycans using recombinant glycosyltransferases expressed extracellularly. These enzymes are typically mutated so that their active site can accommodate bulky substituents. This method, termed one-step selective exoenzymatic labeling (SEEL), was pioneered by the Boons group.^98^ Initial work with SEEL demonstrated improved labeling efficiency and allowed for the enrichment of a large number of glycoproteins. Hong *et al.* employed a similar approach to transfer fucose analogs conjugated with fluorescent dyes directly onto the surface of zebrafish embryos.^99^ These chemoenzymatic methods bypass a sugar's salvage pathway. While this results in robust labeling, it also leads to the loss of valuable information including endogenous enzyme activity and could potentially perturb regular biological functioning. Vocadlo and coworkers have demonstrated that some endogenous glycosyltransferases are tolerant bulky functional groups. Specifically, *O*-GlcNAc transferase (OGT), the enzyme responsible for incorporating GlcNAc on to intracellular proteins, has an incredibly tolerant binding pocket.^61^ So tolerant that GlcNAc molecules functionalized with the small fluorophore 4-nitro-2,1,3-benzoxadiazole (NBD) is accepted as an OGT substrate and added onto proteins.^100^

### 5.3. Multi-labeling

So far, this review has covered singular combinations of reporter-tag ligations. While useful, this approach oversimplifies glycosylation events that are heterogenous in both structure and function. The advent of new bioorthogonal reactions has led to a large enough portfolio of reactive handles that it is now possible to synthesize MCRs with orthogonal reactive groups. These can be administered at once to perform multi-labeling experiments to probe two or more cellular events concurrently without experiencing cross reactivity (Fig. 6C).

The first example of glycan multi-labeling involved co-treating Jurkat cells with Ac_4_ManLev and Ac_4_ManNAz to gain insight into sialic acid metabolism. These results demonstrated a higher turnover to SiaNAz than SiaLev.^101^ Another example comes from the Wittman group who demonstrated concurrent labeling with Ac_4_ManPtl, an alkene functionalized IED-DA probe, and Ac_4_GalNAz.^26^ HeLa cells were grown in the presence of both reporters and co-stained with tetrazine and cyclooctyne conjugates allowing for the detection of both on the cell surface. More recently, Schart *et al.* successfully utilized three orthogonal reactions to triply label HEK293T cells. Mannosamine derivatives, Ac_4_ManNAz, Ac_4_ManAcryl, and Ac_4_ManNCyoc, were able to orthogonally participate in copper-free click chemistry, IED-DA, and photoclick respectively. HEK293T cells treated with all three molecules at once showed independent labeling of each, demonstrating that all three reactions can occur simultaneously with limited background.^102^

Some salvage pathways are so tolerant of structural changes that the same sugar can be functionalized with two different bioorthogonal handles. Feng *et al.* demonstrated this possibility with 9AzSiaNAlk and 9AzSiaNAz.^103^ 9AzSiaNAz has particularly useful applications as a photo-cross linking metabolic reporter. Previously, diazirine functionalized MCRs such as SiaDAz were limited to examining known glycan binding partners that can be readily detected. With 9AzSiaNAz, concurrent incorporation of an azide and diazirine allows for UV-mediated cross-linking products to be identified through biotin enrichment. The initial proof-of-concept study demonstrated that 9AzSiaDAz was able to capture the dimerization of CD22 whereas 9AzSia treated cells only enriched for monomeric CD22.

Arguably the most exciting application of multi-labeling is the potential to probe two separate biological events at once, allowing researchers to answer complicated questions. A seminal example comes from Vocadlo and coworkers. Until recently, *O*-GlcNAc modified substrates have been thought to be modified exclusively post-translationally. However, a study conducted by Zhu *et al.* demonstrated that *O*-GlcNAcylated substrates are modified both post-translationally and co-translationally and that these co-translational modifications have functional roles.^104^ Previous labeling strategies offered no way to selectively enrich for *O*-GlcNAc modifications on nascent peptide chains. Recently, Zhu *et al.* illustrated that tandem labeling could solve this problem.^105^ Cells were treated with two reporters, Ac_4_GalNAz to label *O*-GlcNAc analog and *O*-propargyl-puromycin (OPP) to prematurely truncate polypeptides. Because these reporters bear orthogonal tags, dual enrichment can isolate only double-tagged substrates for subsequent analysis. This strategy allowed the Vocadlo group to identify 175 endogenous nascent peptide *O*-GlcNAc substrates and presented a new covalent labeling technique to isolate and enrich for specific subsets of glycosylated biomolecules.

## 6. Considerations and limitations of MCRs

Despite the simultaneous advancement selective reporters and improved bioorthogonal reactions, MCRs are not perfect. The following section describes some of the major limitations of reporter molecules.

### 6.1. Physiological relevance

Whenever an exogenous species is introduced into a biological environment, the system will experience some sort of perturbance. The extent of this perturbance can convolute the results of the experiment, casting doubt as to the physiological relevance of any observations. This is particularly true for MCRs, which rely on the support of a series of endogenous enzymes for incorporation on proteins or oligosaccharide chains (Fig. 2). The resulting data is interpreted as a metabolic fingerprint that lends insights into the fate of monosaccharide building blocks and functions of labeled glycoproteins.^106^ The major shortcoming of this system is that these unnatural sugars directly compete with native metabolites. Minimally, this means that MCRs turned over by salvage pathway enzymes at the same rate as their analogous sugar moieties disrupt monosaccharide metabolism. More often, MCRs are incorporated less efficiently than their monosaccharide counterparts.^76^ While this means that cellular metabolism may not be as greatly affected, it leads to substoichiometric incorporation that misses valuable information. The Hsieh-Wilson lab developed a chemoenzymatic labeling technique that mitigates this limitation.^107,108^ They engineered a mutant β-1,4-galactosyltransferase enzyme (Y289L GalT) that adds UDP-GalNAz onto endogenous *O*-GlcNAc modifications. This method affords quantitative labeling of *O*-GlcNAcylated proteins. However, chemoenzymatic enrichment is limited to *in vitro* applications in cell lysates or fixed cells due to the requirement of exogenous GalT and UDP-GalNAz. Together, MCR incorporation and chemoenzymatic labeling offer complimentary techniques that can validate results leading to higher confidence in any conclusions.

### 6.2. Selectivity

One of the main objectives when designing monosaccharide MCRs is to establish a degree of selectivity. The ability to predict the fate of an MCR allows researchers to definitively draw conclusions from the results of any given experiment. Though an incredible amount of work has been put toward this goal (see Section 4) it still remains a challenge due to the fact that all mammalian glycans are composed of the same few building blocks (Fig. 1A). For example, GlcNAc is a major component of all the main forms of glycosylation (Fig. 1B). Accordingly, the first reported GlcNAc MCR, Ac_4_GlcNAz, was shown to be incorporated into *N*-linked, *O*-linked, and *O*-GlcNAc modified glycoproteins.^38,39^ Introducing selectivity is further complicated by metabolic cross talk. Endogenous salvage pathway enzymes are able to interconvert monosaccharides and high energy sugar-donors.^38,72,109^ For instance, GlcNAc can be reversibly converted to both GalNAc and ManNAc. This is useful for studying endogenous sugar metabolism.^110^ However, it complicates most applications of MCRs. Fortunately, individual salvage pathways exhibit some amount of substrate selectivity. These inherent differences can be exploited by generating MCRs with subtle structural changes, biasing labeling toward a single type of glycosylation. Our lab has successfully implemented this approach to develop reporters that specifically label *O*-GlcNAc modified proteins^74,75^ which should encourage the development of increasingly diverse MCRs.

**Fig. 7:**
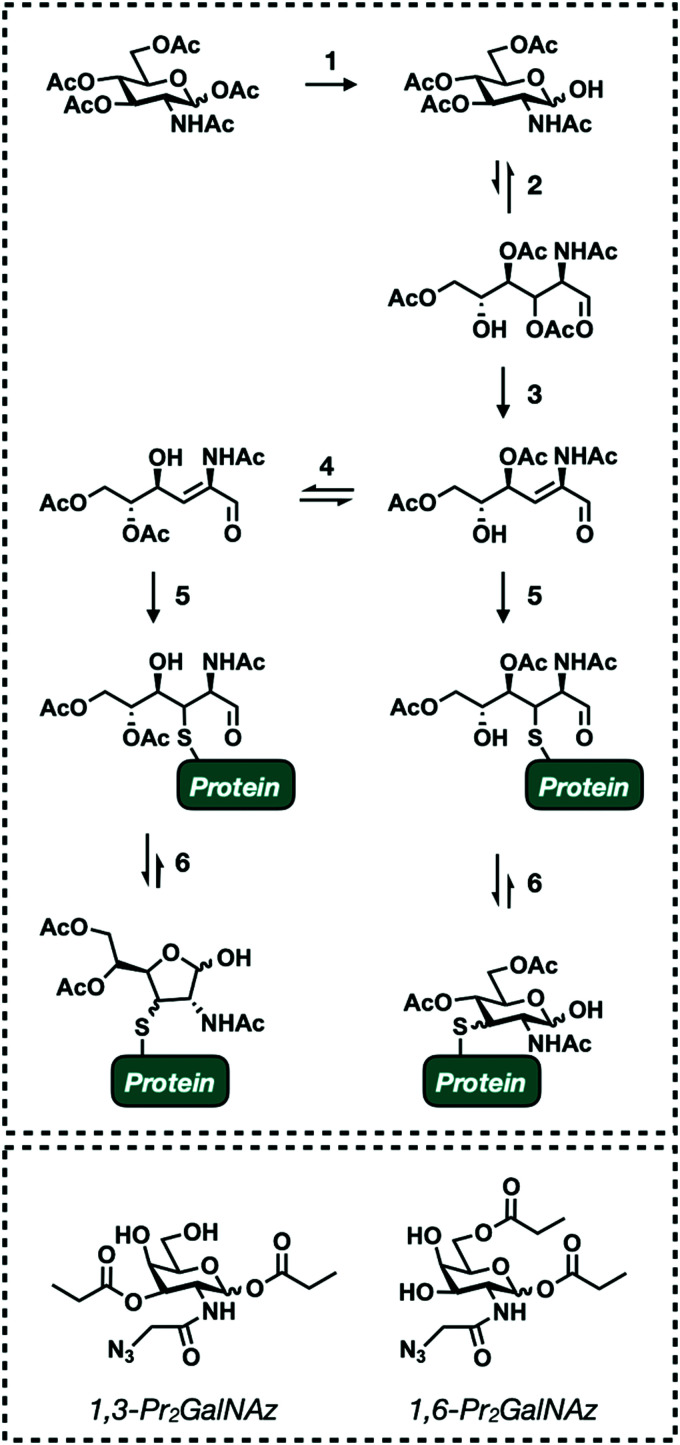
Mechanistic insights into *S*-glycosylation. (top) Proposed mechanism for non-enzymatic *S*-glycosylation. (1) Per-acetylated sugars are deacetylated at the anomeric position. (2) The resulting hemiacetal exists in equilibrium between its open and closed confirmations. (3) Open sugars can undergo β-elimination reaction with a proximal bases. (4) Acyl migration between C4 and C5 position generates two isomers. (5) α,β-Unsaturated aldehydes are susceptible to Michael-addition with endogenous thiols. (6) Ring closure generates 3-thiol furanose and pyranose adducts. (bottom) Structures of next partially acetylated MCRs.

The preceding paragraph describes non-specific enzymatic labeling where the promiscuity of glycosyltransferases and other relevant enzymes leads to unintended incorporation events. A second selectivity issue stems from non-enzymatic chemical labeling of proteins. Chemical labeling was recently discovered as a result of a routine proteomics profiling of HeLa cells with Ac_4_ManNAz.^111^ MCRs are usually administered as their per-acetylated derivatives. This allows for passive diffusion across the cell membrane.^112,113^ Once inside, nonspecific esterase enzymes cleave the acetyl groups to release the reporter molecule (Fig. 2).^114^ Masking the polar hydroxyl groups greatly enhances cellular uptake lending to robust labeling with low treatment concentrations. However, Qin *et al.* identified a significant number of modified cysteine residues in a process termed *S*-glycosylation.^111^ A subsequent mechanistic investigation determined that *S*-glycosylation follows an elimination-addition mechanism facilitated by the presence of acetyl groups on the C2, C3, and C4 positions of the functionalized sugar^111,115^ (Fig. 7). Briefly, per-acetylated sugars are first deacetylated at the anomeric position. The resulting hemiacetal exists in equilibrium between its open and closed confirmations in aqueous environments. When open, the C3 acetyl undergoes base promoted β-elimination to form an α,β-unsaturated aldehyde. This intermediate is then susceptible to Michael-addition with endogenous thiols followed by ring closer to generate 3-thiolated pyranose and furanose adducts. The resulting products are indistinguishable from legitimate enzymatic labeling events without advanced MS-based analysis. The extent of this “off-target” labeling varies by cell type and reporter but has been observed to account for up to 25% of total glycosylation events. Fortunately, lowering the treatment times and concentration when using per-acetylated sugars reduces cysteine labeling.^116^ In an effort to circumvent this issue altogether, Qin *et al.* synthesized two GalNAz derivatives, 1,3-Pr_2_GalNaz and 1,6-Pr_2_GalNAz (Fig. 7). Both demonstrate high labeling efficiency with low background *S*-glycosylation.^115,117^ Both chemical and enzymatic selectivity issues should be taken into consideration whenever performing an experiment with a MCR. Any identified glycoprotein should be validated using an orthogonal technique such as chemoenzymatic enrichment to rule out artificial labeling or unexpected incorporation.

### 6.3. Detection and interpretation of labeled proteins

**Fig. 8:**
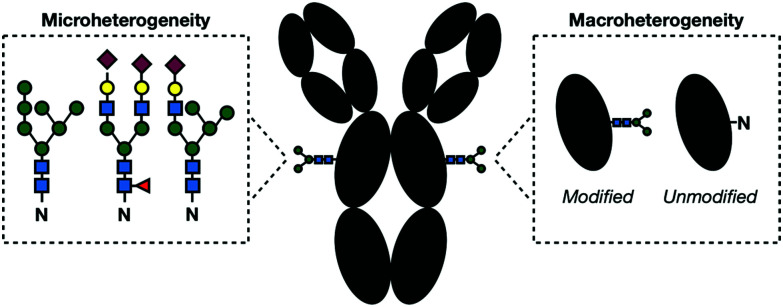
In a given glycoprotein, glycans can vary in their structure and linkages (microheterogeneity) and presence (macroheterogeneity) complicating their detection and identification.

The regulation of glycosylation events is an important part of maintaining proper cellular functions. As such, there has been increasing interest in proteomic profiling to assess a system's overall glycosylation state. Typically, these studies use mass-spectrometry (MS)-based methods which combine sensitive detection with computational identification. To work effectively, a standard MS workflow relies on predictable metabolic structures which will be searched against database algorithms for peptide identification. Unfortunately, glycans are not predictable. In fact, glycosylation has the greatest amount of proteomic diversity compared with all other PTMs. Glycopeptides can vary in both site occupancy (macroheterogeneity) and oligosaccharide structure (microheterogeneity) (Fig. 8).^118^ The resulting heterogenous population of glycans produces a number of substoichiometirc modifications on glycoproteins requiring large amounts of sample to deliver a suitable signal-to-noise ratio. This, in combination with inherently low ionization efficiency of glycoconjugates compared to their unmodified counterparts, makes proper detection of glycoproteins challenging. Even with enough sample, the non-templated nature of glycosylation makes the unambiguous identification of both modification site and context nearly impossible.

With no universal solution, early glycoproteomic investigators adapted a “divide-and-conquer” approach, gathering site and context data in separate MS experiments.^118^ These protocols rely on glycoprotein enrichment strategies to fish out relevant proteins from the mix of modified and unmodified substrates. The emergence of MCRs greatly improved enrichment techniques. Functionalized sugars can be selectively reacted with appropriate chemical partners conjugated to an affinity tag such as biotin. Initial investigations relied on the reduction of glycans to a predictable and uniform mass for proper site identification.^119,120^ These experiments offer binary “yes-or-no” data sets that are missing information associating glycan structure to modification site undermining its biological significance. Woo *et al.* developed a strategy to solve this problem through the invention of isotope-targeted glycoproteomics (IsoTaG).^121,122^ The IsoTaG platform consists the following major components: (1) labeled glycoproteins with MCRs, (2) tagging and enrichment with an isotope recoding affinity probe, and (3) directed tandem MS. Using a dibrominated tag which can be recognized by previously developed IsoStamp software, intact glycopeptides are recognized in real time and selectively submitted to a second ionization event to generate paired glycan and peptide fragments. IsoTaG allows for simultaneous site and context identification. In a proof of concept study, Woo *et al.* labeled Jurkat, PC-3, and MCF-7 cell lines with Ac_4_GalNAz and Ac_4_ManNAz, illustrating that both are incorporated into a range of different glycosylation subtypes.^121^

Even with improvements in MS methods and enrichment techniques, the field of glycoproteomics still has some major challenges. First, IsoTaG and other technologies are only as selective as the chosen MCR. As such, they suffer from the same limitations as those addressed in Section 6.2. This was exemplified in recent work from the Kohler lab.^110^ Here, efforts to generate a probe-free technique for the identification of glycoproteins illuminated the vast metabolic cross talk that interconverts GalNaz, GlcNAz and ManNAz. Additionally, there is still no universal MS workflow that generates site, structure, and biological context in one mega experiment. Native structures, PTM crosstalk, and protein–glycan interactions are commonly lost through the multiple enrichment and digestion steps that yield glycopeptides.

## 7. Conclusions and future outlook

Metabolic chemical reporters revolutionized the study of glycosylation. Improvements in both MCR design and bioorthogonal chemistries have expanded the types of imaging and characterization experiments possible. These advancements allow for *in vivo* studies, offering simple solutions to answer complicated questions. Though limitations exist, advancements in MS-based technology coupled with a greater understanding of monosaccharide metabolism help to solve these problems. The future of MCRs shows great promise in both basic and industrial research. From a fundamental standpoint, MCRs are making great headway toward mapping the total glycome. These findings, in combination with advancements in medicinal chemistry, can advise advancements in pharmaceutical development.

## Conflicts of interest

The authors declare no conflict of interest.

## Supplementary Material
